# Long-term cardiac remodeling associated with heart failure following left-ventricular valve replacement surgery: A retrospective study

**DOI:** 10.1097/MD.0000000000026594

**Published:** 2021-07-30

**Authors:** Mengyuan Chen, Xin Yao, Daowen Wang, Chenze Li, Fei Ma, Rui Li, Hong Wang, Sheng Wei, Qiang Zhou

**Affiliations:** a Division of Cardiology, Department of Internal Medicine, Tongji Medical College, Huazhong University of Science and Technology, Wuhan; b Department of General Medicine, Guangdong Provincial People's Hospital, Guangdong Academy of Medical Sciences, Guangzhou; c Department of Epidemiology and Biostatistics, Ministry of Education Key Laboratory of Environment and Health, School of Public Health, Tongji Medical College, Huazhong University of Science and Technology, Wuhan, China.

**Keywords:** cardiac remodeling, heart failure, valve replacement

## Abstract

To investigate long-term cardiac remodeling and prognosis of patients post-left-ventricular valve replacement, and explored related risk factors of heart failure and management strategies.

Retrospective cohort of patients with left-ventricular valve replacement between 2005 and 2007. Major adverse cardiac events were recorded, including death, hospitalization, stroke, and New York Heart Association (NYHA) functional classifications. Cardiac remodeling was assessed by comparing pre-operative, post-operative, and follow-up echocardiographic images.

(1) Two hundred fifty-seven patients who received left-ventricular mitral, aortic, or double-valve replacement surgery were followed up for 10.4 ± 1.5 years with an all-cause mortality rate 18.7% and an incidence of heart failure that significantly restricted daily life (NYHA III or IV) 21.3%.
(2) There were no significant differences in classic cardiac-remodeling variables between baseline and long-term follow-up, such as left-ventricular diameter (47.9 ± 8.3 vs 49.9 ± 8.0 mm, *P* = .14) and left-ventricular ejection fraction (58.6 ± 9.6% vs 57.0 ± 10.3%, *P* = .34), whereas there were significant differences in terms of left-atrial anteroposterior diameter (LA) (39.7 ± 9.5 vs 49.0 ± 14.3 mm, *P* < .001) and tricuspid regurgitation (TR) (1.4 ± 1.0 vs 2.2 ± 1.2, *P* < .001). Multivariable logistic regression analysis showed that LA ≥ 50 mm (*P* = .011) and more than moderate tricuspid regurgitation (TR > 2) (*P* = .012) were associated with poor prognoses for long-term consequences of heart failure. Both LA and TR progressed with the length of time after surgery.

LA enlargement and TR after left-ventricular valve replacement surgery were time-dependent events, which represented cardiac remodeling and were closely related to post-operative long-term consequences of heart failure. It is important to be cognizant of and to explore long-term preventive and treatment strategies for adverse cardiac events in patients following left-ventricular valve replacement.

## 1 Introduction

Currently, valvular heart disease remains a serious public health issue. The incidence of moderate to severe valvular heart disease with significant clinical presentations is 2.5%.^[1]^ In patients over 65 years old, this incidence is reported to reach 6.4%.^[2]^ Successful valvular surgery may significantly reduce mortality and is the mainstay treatment for these individuals.^[3]^

However, it remains unknown if the benefits of cardiac valvular surgery last long-term. Studies found that post-operative survival rates after mitral-valve surgery were 97.5%, 87.5%, and 43.0% at 5, 10, and 20 years post-surgery, respectively.^[4,5]^ The 15-year survival rate after aortic-valve replacement surgery was 60.6% for biological valves and 62.1% for mechanical vales.^[6]^ A previous study on the long-term prognosis after aortic-valve replacement surgery found that congestive heart failure was an important cause of death (21.0%).^[7]^ One, 5, 10, and 15 years after aortic-valve replacement surgery, freedom of heart failure was 98.6% ± 0.3%, 88.6% ± 1.0%, 73.9% ± 2.3%, and 45.2% ± 8.5%, respectively.^[8]^ These findings suggest that patients carry a risk of poor long-term prognosis even after successful valve replacement surgery.

Although there are many studies on post-operative valvular heart disease, the long-term post-operative prognosis of this cohort has not received adequate attention. Some studies showed improvement of cardiac remodeling after left-ventricular valve replacement surgery, which suggests that this surgical treatment successfully resolves the valvular mechanical lesions and reverses cardiac remodeling.^[9,10]^ However, there remains residual cardiac remodeling after left-ventricular valve replacement surgery, which is associated with poor prognosis.^[10]^ These previous studies have been limited to the assessment of short- and/or medium-term recoveries. In contrast, there have been few studies on the prognosis of patients 10 years after the surgery. Additionally, few studies have systematically compared pre- and post-operative echocardiographic features. Due to a paucity of clinical evidence, clinical guidelines do not mention any long-term prognosis or management strategies following left-ventricular valve replacement surgery.^[11]^ Therefore, to investigate such long-term prognoses, as well as to determine the cardiac structure and functional changes after left-ventricular valve replacement surgery, we conducted a clinical follow-up observational study and collected data from 257 consecutive patients (average follow-up 10.4 ± 1.5 years) who received left-ventricular valve replacement surgery at Tongji Hospital affiliated to Tongji Medical College, Huazhong University of Science and Technology, between 2005 and 2007.

## 2 Methods

### 2.1 Study participants (Fig. 1)

This study was based on a retrospective review of left-ventricular valve replacement.

**Figure 1:**
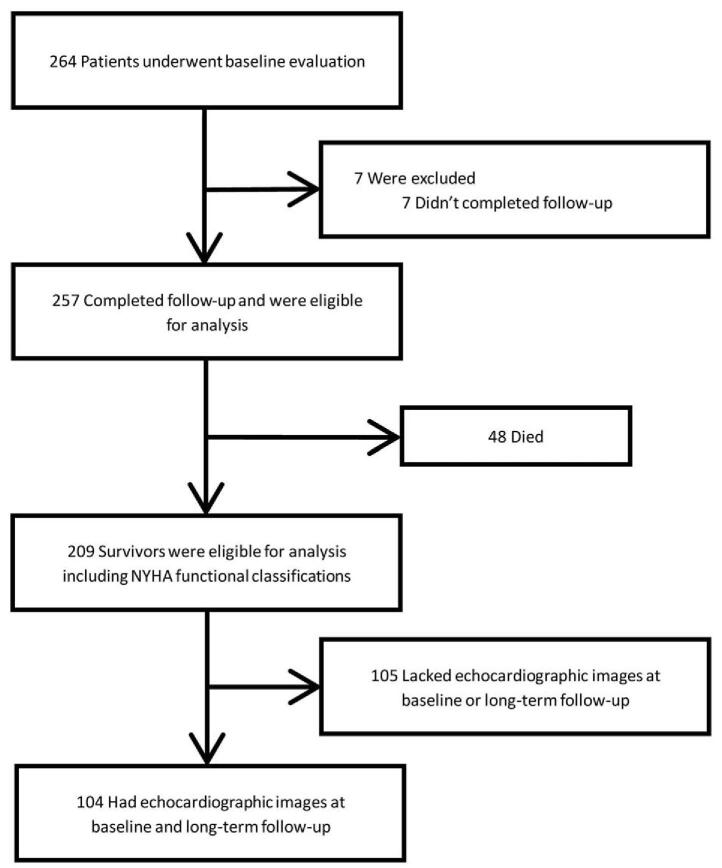
Patient disposition through 10-years follow-up evaluation.

The inclusion criteria were

(1) left-ventricular valve replacement surgery performed between 2005 and 2007 at Tongji Hospital affiliated to Tongji Medical College, Huazhong University of Science and Technology;
(2) left-ventricular valve replacement surgery (mitral, aortic, or double-valve replacements); and
(3) survived until hospital discharge.

The exclusion criteria were

(1) previous operation for left-ventricular valve diseases;
(2) tricuspid valve replacement; and
(3) cardiac transplantation.

The investigation was approved by Tongji Medical College of Huazhong University of Science and Technology Medical Ethics Committee.

### 2.2 Data collection

The data collected included: patient age and gender; re-admission history (e.g., stroke, heart failure, and atrial fibrillation); etiology (e.g., rheumatic heart disease, infective endocarditis, mitral-valve prolapse, congenital heart disease, and nonrheumatic valvular heart disease); comorbidities (e.g., hypertension, hyperlipidemia, diabetes, and coronary heart disease); New York Heart Association (NYHA) functional classification during the follow-up; medications (e.g., anticoagulants, beta-blockers, renin–angiotensin system inhibitors, and diuretics); and echocardiographic images at pre-operative, post-operative, and long-term follow-up periods. Pre- and post-operative baseline echocardiographic images were obtained at 2 weeks before and 2 to 4 weeks after the surgery.

### 2.3 Echocardiography

Patients received echocardiographic examinations during the pre-operative, post-operative, and long-term follow-up visits. Patients were placed in the supine or the left lateral position. Based on the *Guidelines for echocardiographic measurements in Chinese adults*,^[12]^ the anteroposterior diameter of the left atrium, left-ventricular end-diastolic anteroposterior diameter (LVDd), end-diastolic ventricular septum thickness, and left-ventricular posterior wall thickness were measured via parasternal left-ventricular long-axis and short-axis views. The left-ventricular ejection fraction (LVEF) was measured by the dual-plane Simpson method. The peak of the tricuspid regurgitation (TR) spectrum was measured by continuous Doppler at the apex two-chamber and four-chamber views. Pulmonary arterial hypertension can be detected non-invasively with Doppler echocardiography by pulmonary arterial systolic pressure. Right ventricular tricuspid pressure gradient (RVTG) can be considered equal to the pulmonary arterial systolic pressure after adding the estimated mean right atrial pressure of 10 mm Hg.^[13]^

### 2.4 Statistical analysis

Data were analyzed via SPSS 18.0 software (IBM). Continuous data with normal distributions are presented as means ± standard deviations and were compared by independent-sample *t* tests (2 groups) or analyses of variance (ANOVAs; more than 2 groups). Categorical data are presented as percentages (%) and were compared via Chi-square tests. Survival analysis was performed by the Kaplan–Meier method. Multivariable logistic regression analysis was performed to determine prognostic factors. Time-trend evaluations were based on correlational analysis using Spearman correlation.^[14]^ Statistical significance was taken as a *P* value < .05.

## 3 Results

### 3.1 Overall long-term follow-up results after left-ventricular valve replacement surgery (Table 1 and Fig. 2)

A total of 264 patients were received mitral, aortic, or double-valve replacement surgery and discharged alive. Among them, 257 patients (97.4%), with a follow-up period of 10.4 ± 1.5 years were included in the analysis. There were 48 (18.7%) deaths, 17 (8.1%) re-admissions due to heart failure, and 21 (10.0%) re-admissions due to stroke. The numbers and percentages of long-term survivors with different NYHA functional classifications were: grade I, 98 (47.6%); grade II, 64 (31.1%); grade III, 38 (18.4%); and grade IV, 6 (2.9%). There were 44 (21.3%) patients with grade-III and grade-IV NYHA functional classifications that had severe limited daily physical activities. The counts and percentages of patients who received long-term medications were: 108 (96.7%) with warfarin; 28 (13.4%) with beta-blockers; 11 (5.3%) with renin–angiotensin system inhibitors; 14 (6.7%) with loop diuretics; and 19 (9.1%) with the aldosterone antagonist spironolactone.

**Table 1:** Clinical characteristics of study participants.

Variables	Results (N = 257)
Age, yr	51 ± 12.3
Female, N (%)	134 (52.1)
Clinical events, N (%)	
Death	48 (18.7)
Re-admission	102 (48.8)
Stroke	21 (10.0)
Atrial fibrillation	8 (3.8)
Heart failure	17 (8.1)
Etiology, N (%)	
Rheumatic heart disease	172 (66.9)
Infective endocarditis	12 (4.7)
Mitral-valve prolapse	11 (4.3)
Congenital heart disease	8 (3.1)
Nonrheumatic valvular heart disease	54 (21)
Comorbidities, N (%)	
Hypertension	38 (18.2)
Hyperlipidemia	10 (4.8)
Diabetes	12 (5.7)
Coronary heart disease	2 (1)
NYHA classification, N (%)	
I	98 (47.6)
II	64 (31.1)
III	38 (18.4)
IV	6 (2.9)
Medications, N (%)	
Wafarin	202 (96.7)
Anti-platelets	1 (0.5)
Beta-blockers	28 (13.4)
ACEI or ARB	11 (5.3)
Loop diuretics	14 (6.7)
Thiazide diuretics	5 (2.4)
Aldosterone antagonist (antisophthora)	19 (9.1)
Digitalis	22 (10.5)
CCB	21 (10.0)
Antiarrhythmics (Class I+III)	2 (1.0)

**Figure 2:**
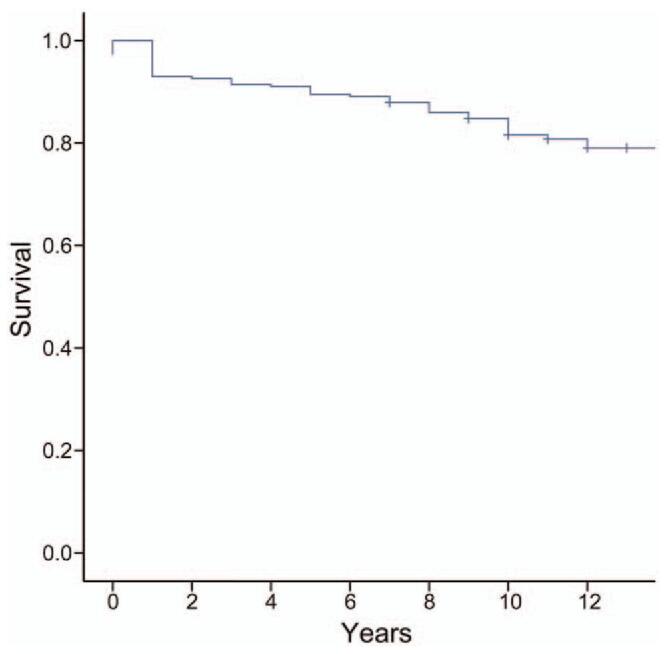
Kaplan–Meier survival analysis showing the long-term survival rate after left-ventricular valve replacement surgery.

### 3.2 Echocardiographic study of long-term cardiac remodeling after left-ventricular valve replacement surgery (Table 2)

To understand long-term cardiac remodeling after left-ventricular valve replacement surgery, we assessed echocardiographic changes from post-surgery baselines (2–4 weeks after surgery) in relation to those at long-term follow-ups. A total of 104 patients had echocardiographic images at baseline and long-term follow-up (7–12 years). There were no statistically significant changes in LVDd (47.9 ± 8.3 vs 49.9 ± 8.0 mm, *P* = .14) or LVEF (58.6 ± 9.6% vs 57.0 ± 10.4%, *P* = .34). In contrast, there were statistically significant changes in left-atrial anteroposterior diameter (LA) (39.7 ± 9.5 vs 49.0 ± 14.3 mm, *P* < .001) and TR grades (1.4 ± 1.0 vs 2.2 ± 1.2, *P* < .001). These results indicate that cardiac remodeling occurred long-term after left-ventricular valve replacement surgery and presented mainly as LA dilatation and TR. After different valve type surgery such as the mitral valve, aortic valve, and double-valve replacement surgery, the long-term progress of LA and TR can still be seen, and the other classic cardiac remodeling indicators have not progressed significantly (Table 2B).

**Table 2:** Comparisons of pre- and post-surgical and long-term follow-up echocardiographic characteristics.

Variables	Pre-operative	Post-operative	Long-term follow-ups	Δ = long-term follow-ups – post-operative	*P* (comparisons between post-operative and long-term follow-ups)
A. Valve types grouped					
LA, mm	48 ± 14.0	39.7 ± 9.5	49.0 ± 14.3	9.34 ± 1.87	<.001^∗^
LVDd, mm	55.7 ± 13.2	47.9 ± 8.3	49.9 ± 8.0	1.98 ± 1.32	.14
IVS, mm	9.83 ± 1.58	9.81 ± 1.53	9.68 ± 1.52	−0.13 ± 0.25	.6
LVPW, mm	9.56 ± 1.67	9.68 ± 1.58	9.6 ± 1.37	−0.08 ± 0.24	.73
LVEF, %	59.04 ± 10.12	58.6 ± 9.6	57.0 ± 10.4	−1.60 ± 1.68	.34
RVTG, mm Hg	43.72 ± 18.55	26.05 ± 8.53	29.55 ± 11.31	3.50 ± 2.77	.21
TR	1.5 ± 1.0	1.4 ± 1.0	2.2 ± 1.2	0.75 ± 0.18	<.001^∗^
TR > 2	7.4%	1.7%	31.7%		<.001^∗^
B. Valve types not grouped					
Mitral-valve replacement					
LA, mm	50.56 ± 13.80	41.59 ± 8.58	50.95 ± 14.76	9.36 ± 2.35	<.001^∗^
LVDd, mm	51.08 ± 10.39	46.26 ± 6.79	49.19 ± 7.44	2.93 ± 1.48	.05
IVS, mm	9.38 ± 1.44	9.28 ± 1.17	9.22 ± 1.14	−0.07 ± 0.24	.78
LVPW, mm	9.04 ± 1.28	9.05 ± 0.94	9.15 ± 1.06	0.10 ± 0.21	.63
LVEF, %	60.13 ± 10.11	59.83 ± 9.21	56.2 ± 10.65	−3.60 ± 2.14	.09
RVTG, mm Hg	41.19 ± 20.17	27.64 ± 8.18	30.51 ± 11.72	2.87 ± 3.35	.40
TR	1.75 ± 0.97	1.54 ± 0.85	2.2 ± 1.13	0.66 ± 0.21	.002^∗^
Aortic-valve replacement					
LA, mm	37 ± 7.39	31 ± 5.48	39.65 ± 8.62	8.65 ± 3.00	.008^∗^
LVDd, mm	68.58 ± 11.29	52.2 ± 9.89	50.95 ± 9.15	−1.25 ± 3.64	.73
IVS, mm	11.16 ± 1.34	11.60 ± 1.71	11.2 ± 1.47	−0.4 ± 0.602	.51
LVPW, mm	11.11 ± 1.66	11.70 ± 2.16	10.95 ± 1.40	−0.75 ± 0.65	.26
LVEF, %	57.50 ± 11.70	55.1 ± 10.42	59.65 ± 7.42	4.55 ± 3.29	.18
RVTG, mm Hg	43.47 ± 6.23	32 ± 7.86	23.85 ± 10.05	−8.15 ± 10.43	.245
TR	0.84 ± 1.02	0.6 ± 0.97	1.45 ± 1.05	0.85 ± 0.40	.04^∗^
Double-valve replacement					
LA, mm	51.17 ± 14.43	40.9 ± 11.69	52.0 ± 14.26	11.1 ± 5.11	.037^∗^
LVDd, mm	55.17 ± 13.60	50 ± 10.85	50.7 ± 8.39	0.7 ± 3.44	.85
IVS, mm	9.75 ± 1.51	10.1 ± 1.20	9.58 ± 1.61	−0.52 ± 0.57	.37
LVPW, mm	9.46 ± 1.74	10.1 ± 1.10	9.58 ± 1.35	−0.52 ± 0.48	.29
LVEF, %	57.68 ± 8.85	57.8 ± 10.09	56.88 ± 11.65	−0.93 ± 4.23	.83
RVTG, mm Hg	50.25 ± 11.84	19.00 ± 7.53	31.06 ± 10.28	12.06 ± 5.48	.04^∗^
TR	1.5 ± 1.0	1.7 ± 0.95	2.63 ± 1.06	0.93 ± 0.39	.02^∗^

### 3.3 Multivariable logistic regression analysis of long-term cardiac dysfunctions after left-ventricular valve replacement surgery (Table 3)

To investigate correlations for the high incidence of long-term cardiac dysfunction after left-ventricular valve replacement surgery, multivariable logistic regression analysis was performed. The incidence of NYHA class III + IV was considered as a poor prognostic index. It was found that gender (*P* = .003), age (*P* = .004), long-term LA enlargement (*P* = .011), and greater than moderate long-term TR (*P* = .012) were associated with poor prognosis of heart failure, whereas classical cardiac remodeling indicators, such as LVDd and LVEF, did not show any correlations (Table 3).

**Table 3:** Logistic regression analysis of variables associated with NYHA classifications.

Variables	HR (95% confidence intervals)	*P*
Female	0.139 (0.037–0.517)	.003^∗^
Age	1.084 (1.026–1.146)	.004^∗^
Valve type	0.492 (0.159–1.518)	.217
LA	1.055 (1.012–1.100)	.011^∗^
TR > 2	1.087 (1.019–1.160)	.012^∗^
LVDd	0.956 (0.851–1.074)	.453
IVS	1.624 (0.411–6.406)	.489
LVPW	0.841 (0.479–1.477)	.547
EF	0.929 (0.848–1.018)	.113
RVTG	1.079 (0.955–1.220)	.223

### 3.4 Correlation analysis of long-term cardiac dysfunctions after left-ventricular valve replacement surgery (Fig. 3)

**Figure 3:**
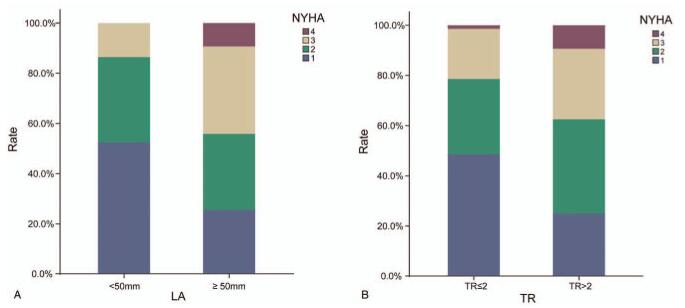
NYHA classifications in patients with varying degrees of long-term LA enlargement and TR following left-ventricular valve replacement surgery. Long-term LA enlargement and TR worsening were associated with higher NYHA classifications, which was significantly different compared to those at baseline. LA = left-atrial anteroposterior diameter; NYHA classification = New York Heart Association function classification.

In order to further clarify the relationship between LA dilatation and TR with heart failure, long-term follow-up echocardiographic data were analyzed. Patients with LA ≥ 50 mm had a higher cardiac function score than did patients with LA < 50 mm (2.3 ± 1.0 vs 1.6 ± 0.7, *P* < .001; Fig. 3A). This result suggested that a larger LA was associated with more severe heart failure and worse outcomes. Patients with more than moderate tricuspid regurgitation (TR > 2) had a higher NYHA functional classification than those of patients with TR ≤ 2 group (2.2 ± 0.9 vs 1.7 ± 0.8, *P* = .011; Fig. 3B). This finding demonstrates that a higher TR grade is associated with more severe heart failure and worse outcomes.

### 3.5 Time-dependent changes in LA and TR after left-ventricular valve replacement surgery (Fig. 4)

**Figure 4:**
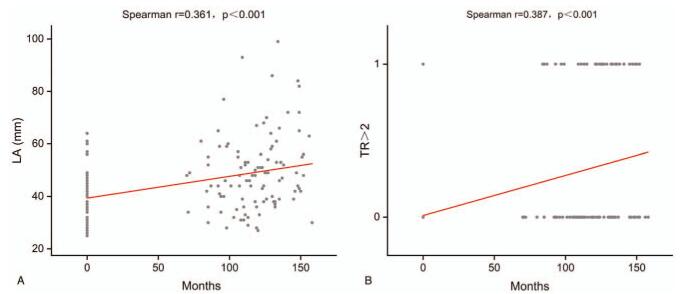
Correlation between cardiac-remodeling variables over time following left-ventricular valve replacement surgery. (A) Correlation of LA with time and (B) correlation of TR > 2 with time. LA = left-atrial anteroposterior diameter; TR > 2 = more than moderate tricuspid regurgitation.

Scatter plots of LA and TR overtime at baseline and at long-term follow-up visits suggested that the progressions of LA enlargement and TR grades (TR > 2) were time-dependent for both (*r* = 0.361, *P* < .001; *r* = 0.387, *P* < .001; Fig. 4A and B). This finding indicates that cardiac remodeling after left-ventricular valve replacement surgery progresses as a function of time from surgery.

## 4 Discussion

The present study found that patients who received left-ventricular valve replacement surgery had unsatisfactory long-term prognosis, with the mortality rate being 18.7% and the incidence of severe heart failure (NYHA functional classifications III + IV) being 21.3%. Additionally, echocardiographic results showed that the classical left-ventricular dysfunction predictors, LVDd and LVEF, were unable to predict long-term consequences of heart failure. Conversely, LA enlargement and TR gradually increased over time post-surgery and were associated with heart failure. Finally, following left-ventricular valve replacement, LA dilatation and TR progressed with time and displayed characteristic changes of long-term cardiac remodeling. After different valve type surgery such as the mitral valve, aortic valve, and double-valve replacement surgery, the long-term progress of LA and TR can still be seen, and the other classic cardiac remodeling indicators have not progressed significantly.

In the study cohort, the 10-year survival rate after left-ventricular valve replacement was 81.3%. Among surviving patients, the proportion of patients with severe cardiac dysfunction was 21.3%. These findings suggest that the long-term prognosis was poor and that the cardiac dysfunction classification was high after left-ventricular valve replacement surgery. In a long-term follow-up study (7.1 ± 2.7 years) in 96 patients with functional tricuspid regurgitation who received mitral-valve replacement surgery, the 5- and 10-year survival rates were 97.5% and 87.5%, respectively.^[4]^ These results suggest that there is still a risk of long-term poor prognosis after successful surgical heart valve replacement surgery.

The main indications for left-ventricular valve replacement surgery are mitral regurgitation and aortic regurgitation with or without aortic stenosis. In addition to ventricular hypertrophy caused by the increased left-ventricular pressure load from aortic stenosis, the main cardiac remodeling changes before valve replacement surgery are secondary to increased left-ventricular volume overload. This can lead to left-ventricular enlargement and EF deterioration from compensation to decompensation, which ultimately results in heart failure with reduced EF. After valve replacement surgery, the valvular mechanical dysfunction is corrected and cardiac enlargement can improve significantly in the short term. However, several studies, including the present study, showed that cardiac remodeling still exists and continues to progress after surgery. This in turn may contribute to poor cardiac function and decreased long-term survival. A meaningful finding from the present study is that classical cardiac remodeling indicators (such as LVDd, EF, and RVTG) do not reliably predict outcomes following valve replacement surgery. On the contrary, the present study showed that the long-term cardiac remodeling pattern after left-ventricular valve replacement was similar to that of heart failure with preserved EF. This suggests that post-operative long-term cardiac remodeling may not be a residual or continuation of valvular heart damage, but is likely the consequence of the valve replacement procedure or from the implanted prosthetic valve. The mechanism of cardiac remodeling after left-ventricular valve replacement has not been fully clarified. Some studies suggested that the anatomical structure and position of the prosthetic valve were different from those of the original valve. These post-surgical deviations may cause hemodynamic dysfunction and/or aberrant blood flow, which have been considered to play an important role in the process of cardiac remodeling.^[15]^ Studies showed that patients with left-ventricular valve repair have a greater survival advantage than patients with replacement surgery. This survival advantage was shown to become more significant over time.^[16]^ These findings highlight the importance of developing improved techniques for performing valve replacement surgery and in improving the selection/use of prosthetic valves.

TR commonly becomes worse with pulmonary hypertension in patients with primary left-ventricular valvular disease. This is well understood since valvular heart disease can cause an increase in left-ventricular pressure and capacity load, which in turn enlarges the LA. This can increase the pulmonary arterial pressure and elevate the right-ventricular load, with the final outcome inducing progressions of TR and pulmonary hypertension. Our present study showed that, after left-ventricular valve replacement surgery, the pre-operative TR and RVTG were significantly decreased and ameliorated. However, the long-term progression of post-operative TR was not accompanied by progression of pulmonary hypertension. The incidences of moderate TR increased from 1.7% in the post-operative baseline to 31.7% in the long-term follow-up period, but the changes in RVTG were not statistically significant. Obviously, this finding cannot be explained simply by right heart failure from blood backflow due to left heart failure. A more likely explanation is that, after left-ventricular valve replacement surgery, long-term left-ventricular remodeling induces right-ventricular remodeling which may promote TR. It has been shown that TR is caused by the expansion of the tricuspid annulus.^[17]^ Tricuspid valve-annulus dilation is recommended as an indication (Class IIa and b) for simultaneous tricuspid valve surgery during left-ventricular valve surgery.^[11,18]^ However, the improvement of TR after tricuspid valve surgery does not completely convert into a survival benefit.^[19]^ Therefore, TR exacerbation is primarily a right-ventricular disorder. Even if surgery corrects TR, it does not fundamentally improve the prognosis of right-ventricular dysfunction. This also suggests that the mechanism and pattern of cardiac remodeling after left-ventricular valve replacement surgery are different from those of primary valvular disease.^[20]^ Nardi et al investigated a total of 108 patients who underwent left atrial ablation by means of unipolar or bipolar radiofrequency for atrial fibrillation in association with mitral or mitral and aortic valve surgery. The study showed that the left atrium size is associated with poor long-term prognosis after left-ventricular valve replacement.^[21]^

Clinic data including present study shows that cardiac remodeling persists even after left-ventricular valve replacement surgery, which leads to poor long-term outcomes. This understanding reminds us to carefully manage patients after valve replacement surgery. The latest 2014 AHA/ACC and 2017 ESC/EACTS guidelines for valvular heart disease do not mention any drug therapies for cardiac remodeling after cardiac valve replacement surgery.^[11,18]^ In clinical practice, most patients do not receive long-term medication (except warfarin) after successful valve replacement surgery. In our present study, up to 96.7% of patients still took warfarin 10 years after their surgery. Traditionally, β-blockers, angiotensin converting enzyme inhibitor and/or angiotensin II receptor blocker, and aldosterone inhibitors that can reverse or delay cardiac remodeling are not used in the clinic. This might be related to the long-term cardiac dysfunction found in our present study population. However, results herein suggest that the cardiac remodeling pattern after left-ventricular valve replacement surgery is different from that of the traditional heart failure with reduced EF model. The effectiveness of above drug treatments on preventing long-term cardiac remodeling following left-ventricular valve replacement surgery remains to be determined.

## 5 Limitations

The limitations of the present study include that it was a single-center study with a small number of patients. Only 104 (40.5%) patients had complete echocardiographic images available for analysis. Also, the retrospective study design of our present study had inherent biases. Due to the small sample size, the correlation is not strong, but the long-term cardiac remodeling collected by echocardiography can reflect the trend of progress over time. In the future, we can collect short-term, mid-term, and long-term continuous echocardiography data after surgery to better prove the time trend. We did not fully consider the inclusion and exclusion criteria, especially with respect to atrial flutter or fibrillation, AV conduction defects. We did not use the right heart catheter to measure pulmonary arterial pressure and pulmonary impedance accurately. Echocardiographic estimates of pulmonary arterial pressure may be less accurate. Future multi-center studies with larger patient populations and complete data sets are required to validate the present results.

## 6 Conclusions

In the present study, it was found that left atrial enlargement and tricuspid regurgitation progressed over time and represented characteristic changes of cardiac remodeling after left-ventricular valve replacement surgery. These changes were closely related to long-term heart failure after surgery. Hence, more studies are warranted to investigate long-term prevention and treatments after left-ventricular valve replacement surgery.

## Author contributions

**Conceptualization:** Qiang Zhou.

**Data curation:** Mengyuan Chen, Chenze Li, Qiang Zhou.

**Formal analysis:** Mengyuan Chen, Sheng Wei, Qiang Zhou.

**Investigation:** Mengyuan Chen, Xin Yao, Fei Ma, Rui Li, Hong Wang, Qiang Zhou.

**Methodology:** Mengyuan Chen, Qiang Zhou.

**Project administration:** Daowen Wang, Qiang Zhou.

**Resources:** Qiang Zhou.

**Writing – original draft:** Mengyuan Chen, Qiang Zhou.

**Writing – review & editing:** Mengyuan Chen, Qiang Zhou.
